# Factors associated with alteration of nipple or skin sensation and impact of duration of time following nipple-sparing mastectomy (NSM): an analysis of 460 cases with comparison of conventional versus endoscopic- or robotic-assisted NSM

**DOI:** 10.1186/s12957-023-03107-5

**Published:** 2023-07-26

**Authors:** Hung-Wen Lai, Yi-Lin Chang, Korawan Chandrachamnong, Mee-Hoong See, Hsin-I Huang, Shih-Lung Lin, Dar-Yuan Fang, Shou-Tung Chen, Dar-Ren Chen, Chi Wei Mok, Fiona Tsui-Fen Cheng

**Affiliations:** 1 Endoscopic & Oncoplastic Breast Surgery Center, Changhua Christian Hospital, Changhua, Taiwan; 2 Department of Surgery, Division of General Surgery, Changhua Christian Hospital, Changhua, Taiwan; 3 Comprehensive Breast Cancer Center, Changhua Christian Hospital, Changhua, Taiwan; 4 Minimal Invasive Surgery Research Center, Changhua Christian Hospital, Changhua, Taiwan; 5 Kaohsiung Medical University, Kaohsiung, Taiwan; 6 Division of Breast Surgery, Yuanlin Christian Hospital, Yuanlin, Taiwan; 7 School of Medicine, Chung Shan Medical University, Taichung, Taiwan; 8 School of Medicine, National Yang Ming Chiao Tung University, Taipei, Taiwan; 9 Department of Surgery, Division of Breast Surgery, Police General Hospital, Bangkok, Thailand; 10 Department of Surgery, Faculty of Medicine, Breast Oncoplastic Surgery Unit, University Malaya, Kuala Lumpur, Malaysia; 11 Department of Information Management, National Sun Yat-Sen University, Kaohsiung, Taiwan; 12 Wesing Breast Hospital, Kaohsiung, Taiwan; 13 Department of Surgery, Division of Plastic and Reconstructive Surgery, Changhua Christian Hospital, Changhua, Taiwan; 14 Department of Surgery, Division of Breast Surgery, Changi General Hospital, Singapore, Singapore; 15 SingHealth Duke-NUS Breast Centre, Singapore, Singapore; 16 Department of Surgery, Division of General Surgery, Shin Kong Wu Ho-Su Memorial Hospital, Taipei, Taiwan; 17 School of Medicine, College of Medicine, Fu Jen Catholic University, Taipei, Taiwan

**Keywords:** Nipple-sparing mastectomy (NSM), Nipple sensation, Skin sensation, Nipple areola complex (NAC), Breast cancer, Conventional nipple-sparing mastectomy (C-NSM), Endoscopic-assisted nipple-sparing mastectomy (E-NSM), Robotic-assisted nipple-sparing mastectomy (R-NSM)

## Abstract

**Background:**

The current study aims to evaluate the nipple and skin sensation following nipple-sparing mastectomy (NSM) and identify patient-, surgical-, or treatment-related factors affecting nipple or skin sensation in this cohort.

**Methods:**

Patients who received NSM with postoperative nipple and skin sensation test evaluation at a single institution over the past 10 years were retrospectively retrieved from a prospectively collected breast cancer surgery database.

**Results:**

A total of 460 NSM procedures were included in this current study, with the mean age of 48.3 ± 9.1. Three-hundred eighty-three (83.3%) patients had breast reconstructions. One-hundred seventy-four (37.8%) received conventional NSM (C-NSM), 195 (42.4%) endoscopic-assisted NSM (E-NSM), and 91 (19.8%) robotic-assisted NSM (R-NSM) procedures. For nipple sensation assessment, 15 (3.3%) were grade 0, 83 (18.2%) grade I, 229 (49.7%) grade II, and 133 (28.9%) grade III (normal sensation), respectively, with mean grade score of 2.1 ± 0.7. The preserved (grade III) nipple sensation rate was 36.2% (63/174) in the C-NSM group, 26.7% (52/195) in the E-NSM group, and 19.7% (18/91) in the R-NSM group (*P* = 0.06). The “time since surgery to last evaluation” was significantly longer in the C-NSM group (45.6 ± 34 months) or E-NSM group (44.7 ± 35.8 months) as compared to R-NSM group (31.8 ± 16 months, *P* < 0.01). In multivariate analysis, peri-areolar incision showed higher grade of nipple sensation (*OR*: 2.1, *P* = 0.02) compared to upper outer quadrant incision, and longer follow-up time post-NSM showed significant improvement of nipple or skin sensation (> 60 months vs. ≦ 12 months: nipple odds ratio (OR) = 5.75, *P* < 0.01; skin, *OR* = 1.97, *P* < 0.05).

**Conclusion:**

Our current analysis showed some factors to be related to postoperative nipple or skin sensation, and longer “time after surgery” was associated with significant improvement of nipple and skin sensation in patients who received NSM, regardless of the surgical approaches.

**Synopsis:**

Our current analysis showed a significant portion of patients with decrease or loss of nipple or skin sensation after nipple-sparing mastectomy (NSM). Several factors associated with preserved nipple or skin sensation were identified, including age, surgical methods, surgical wound location, and association of time from surgery showing that improvement of partial nipple or skin sensation was evident after a longer follow-up.

## Highlights

• Diminished or loss of nipple sensation had been reported in 10–75% of patients who received nipple-sparing mastectomy (NSM).

• “Time from surgery to last evaluation” was a significant factor associated with sensation recovery or preservation in nipple or skin sensation post NSM.

• Skin incisions and types of operations (conventional versus endoscopic or robotic assisted NSM) were highly correlated with nipple and skin sensation post-NSM.

• Nipple ischemia necrosis grading was unrelated to preserved or lost nipple sensation post-NSM.

• Post-mastectomy radiotherapy was not associated with nipple or skin sensation post-NSM.

## Introduction

Compared with skin-sparing mastectomy (SSM), patients who received nipple-sparing mastectomy (NSM) showed significantly improved aesthetic outcomes and quality of life (QoL) [1–3]. NSM is now increasingly adopted as one of the standards for patients undergoing mastectomy without nipple-areolar complex (NAC) involvement due to acceptable oncologic safety [4–6].

Diminished or loss of nipple sensation had been reported in 10–75% of patients post-NSM [7–10]. For this patient group, losing their nipple sensation despite NAC preservation would result in lower satisfaction and QoL than patients who received breast-conserving surgery [11].

Novel surgical techniques of NSM, like endoscopic- or robotic-assisted NSM (R-NSM), had shown a decreased risk of nipple ischemia necrosis [12, 13]. There has been a paucity of studies reporting on the effect of different surgical approaches on nipple or skin sensation post-surgery due to the relatively recent advent of these surgical methods in the past decade. One recently reported randomized controlled trial showed R-NSM to be significantly associated with better nipple sensation compared with conventional nipple-sparing mastectomy (C-NSM) [14]. Further studies or investigations on whether these novel NSM techniques result in better preservation of nipple or skin sensation compared with C-NSM is of utmost importance.

Compared to other research areas of NSM studies, sensation preservation or related factors were underreported. Some studies have reported nipple sensation loss post-NSM, but they were limited to a small number of patients which further limit risk factors analysis of nipple or skin sensation loss [14, 15]. Furthermore, it remains unclear whether the duration of “time since surgery” would result in the recovery of nipple or skin sensation [16, 17].

The aims of the current study were to evaluate the nipple and skin sensation status post-NSM and compared the impact of different surgical approaches, like robotic — versus endoscopic — or conventional NSM on sensation preservation. Further evaluation on the influence of “time since surgery” on the sensation of the nipple or skin and related risk factors would be performed as well.

## Materials and methods

### Patients

Patients who received NSM at Changhua Christian Hospital (CCH) from August 2011 to April 2022 were retrospectively retrieved from a prospectively collected breast cancer surgery database. Patients were invited to receive nipple and skin sensation tests during regular follow-up. Those without detailed clinicopathologic information or nipple and skin sensation evaluations were excluded. This study was approved by the Institutional Review Board of CCH (CCH IRB no.: 190414). All patients provided written informed consent.

Clinicopathologic factors, surgical approaches (C-NSM, E-NSM, or R-NSM), types of breast reconstructions, and surgical wound incisions were recorded. Degrees of nipple or skin sensation post-NSM were assessed during outpatient follow-up. The factors related to loss or preservation of nipple or skin sensation were identified. Patients who had received nipple or skin sensation tests more than once were used to test the recovery curve of nipple or skin sensation post-operation in a longitudinal follow-up.

**Fig. 1:**
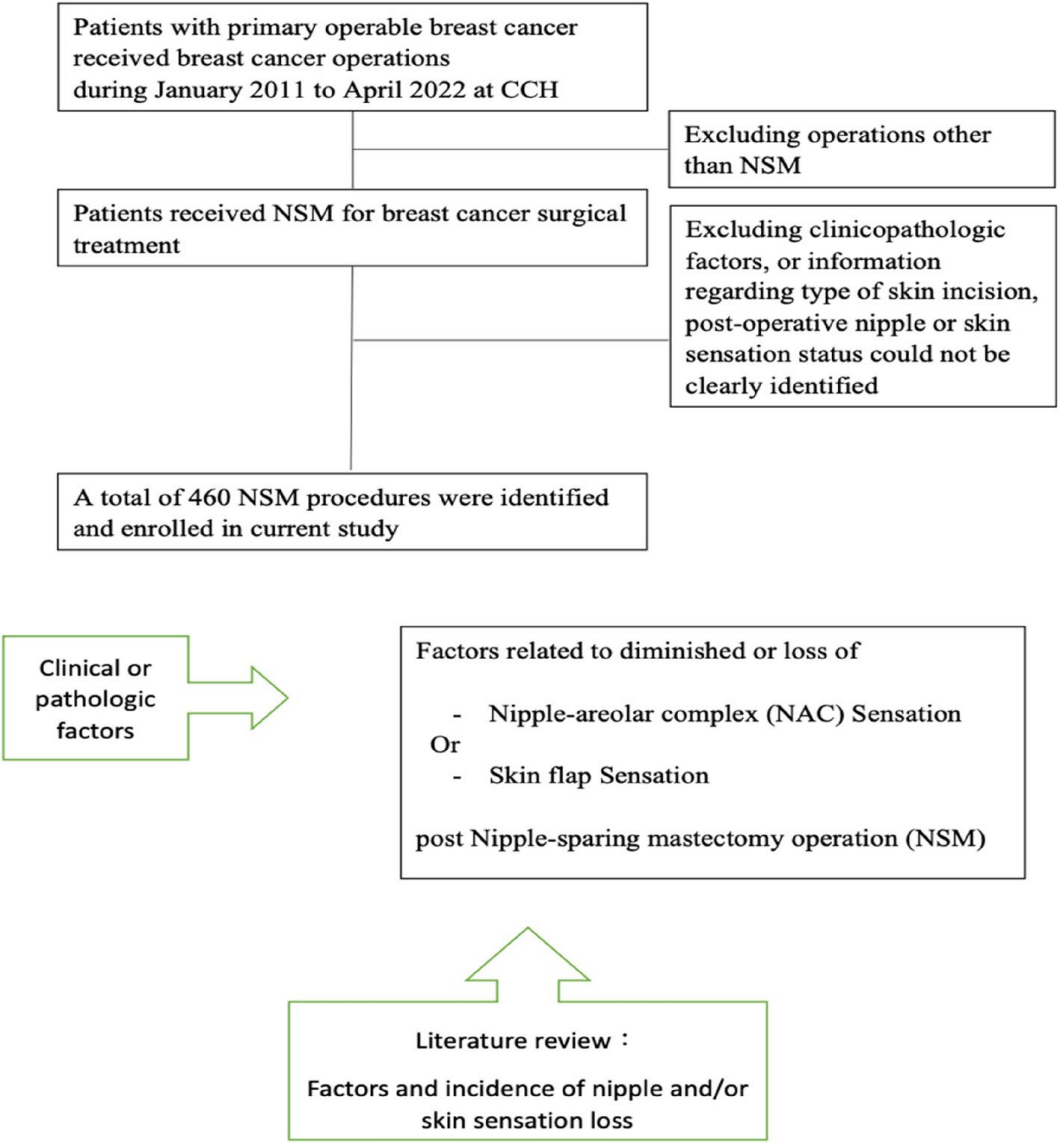
Flow chart of study design. Patients’ enrollment and section criteria. Factors related to loss or preserved sensation of NAC sensation and literature review. NSM, nipple-sparing mastectomy; NAC, nipple areolar complex

The current research also performed a literature review of reported studies [1, 9, 13, 18–22] regarding nipple or skin sensation post-NSM. The study design and patient allocations are shown in Fig. 1.

### Indications of NSM

NSM was indicated for breast cancer patients who opted for mastectomy but keen to preserve their NAC. Patients selected for NSM should have no gross involvement of NAC as evaluated preoperatively through clinical examinations and imaging studies (mammography, sonography, with or without breast magnetic resonance imaging). Patients with nipple involvement reported during intraoperative frozen sections were excluded from inclusion into the current study as the procedure was changed to SSM instead.

### Nipple and skin sensation assessment

**Fig. 2:**
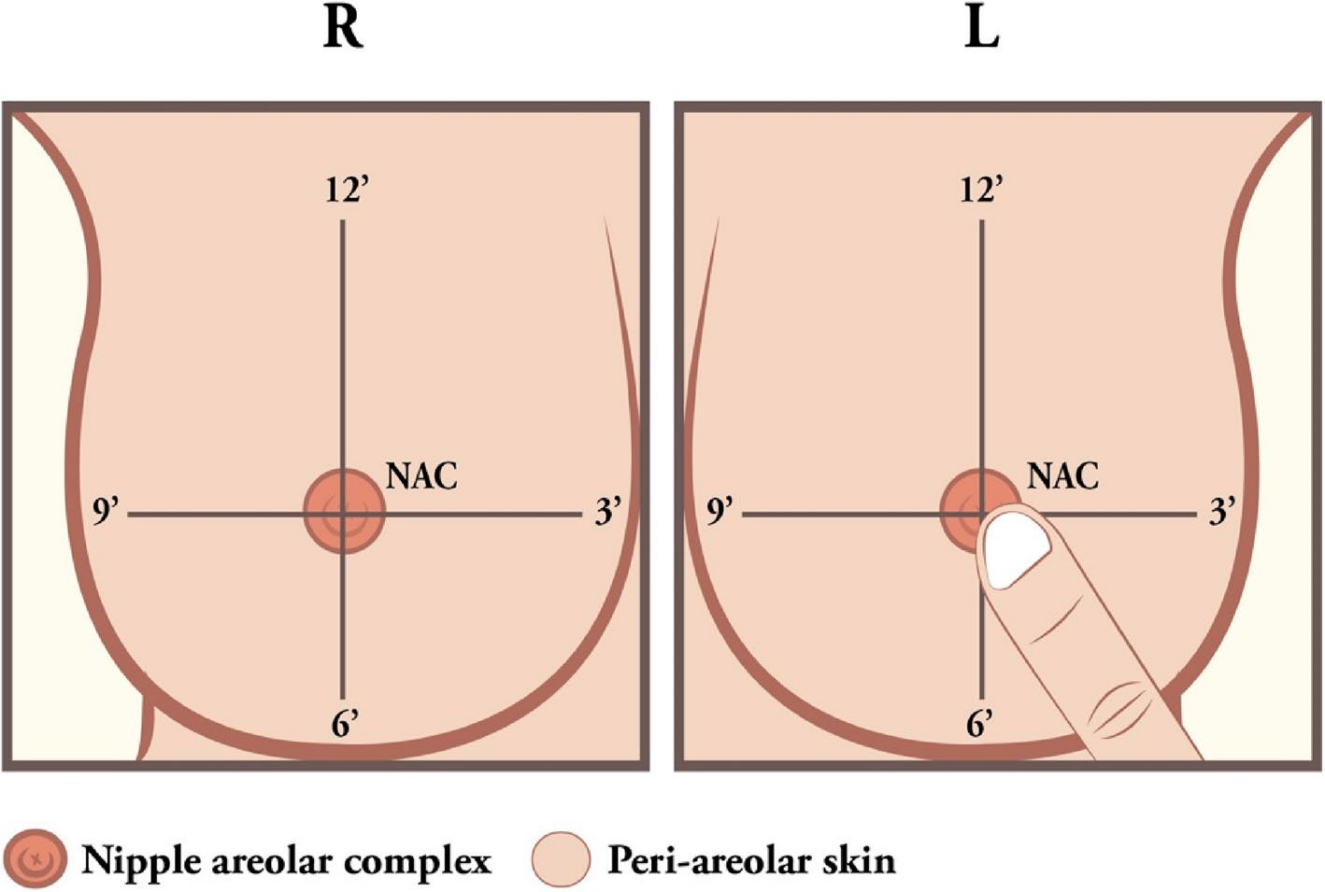
Nipple and breast skin sensation test. The sensation assessment was first evaluated by finger touching the healthy side to show the feeling of touch sensation of a normal breast. Then perform the same touch on the operative side of the nipple and skin. Patients were asked to answer score of sensation when feeling the touch of the nipple or area of skin during an examination. Five area measurement points are as follows: the nipple, upper breast skin, medial breast skin, inferior breast skin, and lateral breast skin were tested, and the nipple or breast skin sensation were divided into four degrees (0–3): grade 0 (no sensation at all), grade I (numbness sensation), grade II (fairly sensate), and grade III (normal sensate)

Patients who received NSM were followed up at the outpatient clinic and invited to have nipple and skin sensation tests by clinical study nurses according to the study protocol. The sensation assessment was first performed with finger touching the healthy contralateral side to demonstrate touch sensation of a normal breast. The same was then repeated on the nipple and skin of the operated side. Patients were then asked to score their sensation to touch of the nipple or area of the skin during an examination. Five areas of the measurement points were as follows: nipple, upper breast skin, medial breast skin, inferior breast skin, and lateral breast skin. Grading of the nipple or breast skin sensation was divided into four degrees (grade 0: no sensation at all, grade I: numbness sensation, grade II: fairly sensate, and grade III: normal sensation, Fig. 2).

### Factors associated with nipple or skin sensation

Factors associated with the change in nipple sensation were identified by comparing patients with preserved nipple or skin sensation versus patients with decreased or lost nipple or skin sensation. Potential factors evaluated include patient-related characteristics, surgery-related factors, postoperative factors, and follow-up duration (time of operation to last performance of nipple or skin test). Patients were divided into retained nipple or skin sensation (grade III) versus diminished or loss sensation groups (grades II, I, and 0).

### Type of skin incisions and operation methods

**Fig. 3:**
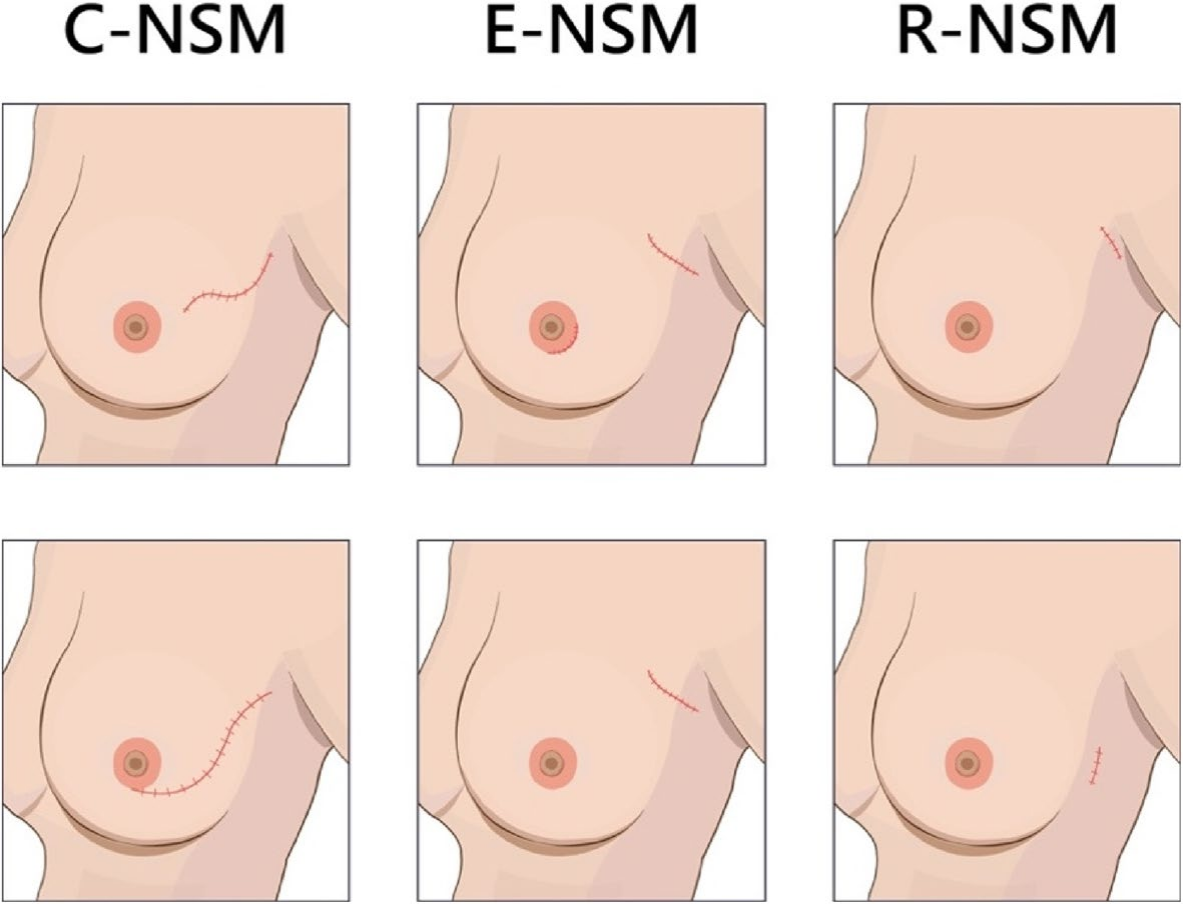
Operation methods and skin incision types. Common skin incisions used in the current study were upper-outer-oblique (radial), peri-areolar, single axillary, or lateral chest incisions. Operation methods for NSM used in the current study included conventional NSM (C-NSM), endoscopic-assisted NSM (E-NSM), or robotic-assisted NSM (R-NSM). C-NSM is usually performed by upper outer oblique (radial) incision or sometimes peri-areolar incision. Some E-NSM patients received dual-axillary-areolar incisions (categorized as peri-areolar incision) or single axillary or lateral chest incisions. R-NSM usually adopts a single axillary incision or lateral chest incision

Common skin incisions used in the current study were upper-outer-oblique (radial), peri-areolar, single axillary, or lateral chest incisions. Operation methods for NSM used in the current study consisted of C-NSM, E-NSM, or R-NSM. C-NSM is usually performed via upper outer oblique (radial) incision or sometimes peri-areolar incision. Some E-NSM patients received dual-axillary-areolar incisions (categorized as peri-areolar incision) or single axillary or lateral chest incisions. R-NSM usually adopted a single axillary incision or lateral chest incision. The type of incisions and operation methods is as shown in Fig. 3.

### Statistical analysis

Baseline data were analyzed using chi-square tests (categorical data) or Student’s *t*-test (continuous data). The chi-square test analyzes the association between breast skin sensation and risk factors. All enrolled patients would receive at least one nipple or skin sensation evaluations, and for patients with more than one nipple and skin sensation assessment, the final evaluation score was used for the final risk factor evaluation analysis. Multivariate logistic regression was performed to assess the association of nipple or skin sensation with other factors. Results were considered statistically significant if the two-tailed *P*-value was < 0.05 for all tests. Statistical analysis was performed using SAS 9.4 version (SAS Inc., Cary, NC, USA).

## Results

A total of 460 NSM procedures were enrolled in the current study, and their mean age was 48.3 ± 9.1 years old. There was 383 (83.3%) immediate breast reconstruction, and 77 cases (16.7%) were without breast reconstructions. One-hundred seventy-four (37.8%) received C-NSM, 195 (42.4%) E-NSM, and 91 (19.8%) were R-NSM procedures. The mean body mass index (BMI) was 23.7 ± 3.7, and 116 (27.1%) received radiotherapy after surgery. The clinicopathologic factors were summarized in Table 1.

**Table 1:** Clinicopathologic characteristics of 460 nipple-sparing mastectomy procedures

	***N* = 460**
**Age, year**	48.3 ± 9.1
< 40	75 (16.3)
≧ 40, < 60	329 (71.5)
≧ 60	56 (12.2)
**Location**	
Left	221 (48.1)
Right	239 (51.9)
**BMI (body mass index)**	23.7 ± 3.7
< 18	19 (4.1)
18–24	271 (58.9)
> 24	170 (37.0)
**Surgical methods**	
Conventional NSM	174 (37.8)
Endoscopic-assisted NSM	195 (42.4)
Robotic-assisted NSM	91 (19.8)
**Specimen size, g (*NA* = 48)**	327.1 ± 184.5
< 180	66 (16.01)
180–320	173 (41.99)
320–450	93 (22.57)
> 450	80 (19.41)
**Surgical ALN staging method (*NA* = 46)**	
SLNB	298 (71.9)
SLNB + ALND	85 (20.6)
ALND	31 (7.5)
**Tumor size, cm (*NA* = 87)**	2.5 ± 2.2
**ALN metastasis on pathology (*NA* = 44)**	
N0	290 (69.7)
N1	96 (23.1)
N2	24 (5.8)
N3	6 (1.4)
**Stage (*NA* = 64)**	
0	87 (21.9)
I	107 (27.1)
II	157 (39.7)
III	44 (11.1)
IV	1 (0.2)
**ER (*NA* = 76)**	
Positive	320 (83.3)
Negative	64 (16.7)
**PR (*NA* = 78)**	
Positive	270 (70.7)
Negative	112 (29.3)
**HER-2 (*NA* = 131)**	
Positive	80 (24.3)
Negative	249 (75.7)
**Ki-67 (*NA* = 189)**	
≦ 14	139 (51.3)
> 14	132 (48.7)
**Pathology (*NA* = 3)**	
DCIS	94 (20.6)
IDC	261 (57.1)
ILC	16 (3.5)
LCIS	3 (0.6)
Other	83 (18.2)
**Operation time**	224.2 ± 124.3
**Reconstruction**	
Yes	383 (83.3)
No	77 (16.7)
**Complication**	
Yes	102 (22.2)
No	358 (77.8)
**Chemotherapy (*NA* = 31)**	
Yes	174 (40.6)
No	255 (59.4)
**Radiotherapy (*NA* = 31)**	
Yes	116 (27.1)
No	313 (72.9)
**Recurrence**	
Yes	37 (8)
No	423 (92)
**Metastasis**	
Yes	30 (6.5)
No	430 (93.5)
**Follow-up time (time since surgery) month**	42.4 ± 32.5

*BMI* body mass index, *ALN* axillary lymph node, *SLNB* sentinel lymph node biopsy, *ALND* axillary lymph node dissection, *NSM* nipple-sparing mastectomy. ★IDC, IDC, IDC + ILC, IDC + LCIS, IDC + DCIS < 10%, IDC + DCIS (IDC + DCIS = IDC + DCIS, IDC + DCIS + LCIS). ★ILC, ILC, ILC + LCIS, ILC + DCIS. ★other, other cancer

All NSM procedures with nipple sensation assessed were evaluated at a mean of 42.4 ± 32.5 months post-operation (time since surgery). Fifteen (3.3%) cases were graded as 0, 18.2% (83/460) grade I, 49.7% (229/460) grade II, and 28.9% (133/460) grade III. The overall mean score of nipple sensation was 2.1 ± 0.7, and the average grade score of skin sensation over four quadrants was 2.1 ± 0.7 (Table 2) as well.

**Table 2:** Surgical methods and surgical wound location related to nipple areolar complex or sensation test

Sensation	Surgical methods				*p*-value
	All (*n* = 460)	C-NSM (*n* = 174)	E-NSM (*n* = 195)	R-NSM (*n* = 91)	
Nipple sensation					0.06
0	15 (3.3)	7 (4.0)	5 (2.6)	3 (3.3)	
1	83 (18.2)	22 (12.7)	41 (21.0)	20 (22.0)	
2	229 (49.7)	82 (47.1)	97 (49.7)	50 (66.0)	
3	133 (28.9)	63 (36.2)	52 (26.7)	18 (19.7)	
Follow-up time (time since surgery), month	42.4 ± 32.5	45.6 ± 34#	44.7 ± 35.8#	31.8 ± 16	< 0.01
NAC sensation average value	Mean, *SD* 2.1 ± 0.7				
	Surgical wound location				*p*-value
Sensation	Upper outer (radial + other) (*n* = 71)	Peri-areolar (*n* = 156)	Single axillary or lateral chest (*n* = 215)	Inframammary fold (*n* = 18)	
Nipple sensation					0.04
0	2 (2.8)	5 (3.2)	8 (3.7)	0	
1	16 (22.5)	22 (14.1)	45 (20.9)	0	
2	36 (50.7)	69 (44.2)	112 (52.1)	12 (66.7)	
3	17 (23.9)	60 (38.5)	50 (23.3)	6 (33.3)	
Follow-up time (time since surgery), month	54.6 ± 32.7*	54.2 ± 37.8*	29.63 ± 20.6	44.8 ± 43.6	0.01
Sensation	Surgical methods				*p*-value
Skin sensation	All (*n* = 460)	C-NSM (*n* = 174)	E-NSM (*n* = 195)	R-NSM (*n* = 91)	
Upper breast					0.17
0	17 (3.7)	5 (2.9)	8 (4.1)	4 (4.4)	
1	68 (14.8)	22 (12.6)	30 (15.4)	16 (17.6)	
2	217 (47.2)	73 (42.0)	97 (49.7)	47 (51.6)	
3	158 (34.6)	74 (42.5)	60 (30.8)	24 (26.4)	
Mean, SD	2.1 ± 0.8				
Lateral breast					0.05
0	22 (4.5)	5 (2.9)	11 (5.6)	6 (6.6)	
1	77 (16.7)	25 (14.4)	30 (15.4)	22 (24.2)	
2	219 (47.6)	78 (44.8)	97 (49.8)	44 (48.4)	
3	142 (30.9)	66 (37.9)	57 (29.2)	19 (20.9)	
Mean, SD	2 ± 0.8				
Inferior breast					0.21
0	18 (3.9)	6 (3.5)	10 (5.1)	2 (2.2)	
1	59 (12.9)	20 (11.5)	25 (12.8)	14 (15.4)	
2	215 (46.7)	72 (41.4)	95 (48.7)	48 (52.7)	
3	168 (36.5)	76 (43.6)	65 (33.3)	27 (29.7)	
Mean, SD	2.1 ± 0.8				
Medial breast					0.62
0	15 (3.3)	6 (3.5)	6 (3.1)	3 (3.3)	
1	55 (12)	20 (11.5)	22 (11.3)	13 (14.3)	
2	214 (46.5)	72 (41.4)	98 (50.2)	44 (48.3)	
3	176 (38.2)	76 (43.)	69 (35.4)	31 (34.1)	
Mean, SD	2.1 ± 0.8				
Skin sensation average value	Mean, *SD* 2.1 ± 0.7				

*NSM* nipple-sparing mastectomy, *C-NSM* conventional NSM, *E-NSM* endoscopic-assisted NSM, *R-NSM* robotic-assisted NSM, *SD* standard deviation, *NAC* nipple areolar complex. #The “time since surgery to last evaluation” was not significantly different in the C-NSM group (45.6 ± 34 months) as compared to E-NSM group (44.7 ± 35.8 months, *P* = 0.79). *The “time since surgery to last evaluation” was not significantly different in the upper outer (radial) incision (54.6 ± 32.7 months) as compared to peri-areolar incision (54.2 ± 37.8, *P* > 0.05)

#The “time since surgery to last evaluation” was not significantly different in the C-NSM group (45.6 ± 34 months) as compared to E-NSM group (44.7 ± 35.8 months, *P* = 0.79). *The “time since surgery to last evaluation” was not significantly different in the upper outer (radial) incision (54.6 ± 32.7 months) as compared to peri-areolar incision (54.2 ± 37.8, *P* > 0.05)

In analysis of different surgical approaches, preserved or normal (grade III) nipple sensation rate was 36.2% (63/174) in C-NSM group, 26.7% (52/195) in E-NSM group, and 19.7% (18/91) in the R-NSM group (*P* = 0.06, Table 2). It has to be noted that “time since surgery to last evaluation” was noted to be significantly longer in the C-NSM group (45.6 ± 34 months) or E-NSM group (44.7 ± 35.8 months) as compared to R-NSM group (31.8 ± 16 months, *P* < 0.01). (C-NSM group (45.6 ± 34 months) compared with E-NSM group (44.7 ± 35.8 months) was not statistically significant, *P* = 0.79.) In terms of surgical wound placement, preserved nipple sensation was noted to be 23.9% (17/71) in the upper outer oblique (radial) incision group (time since surgery: 54.6 ± 32.7 months), 38.5% (60/156) in the peri-areolar-related incision group (54.2 ± 37.8 months), 33.3% (6/18) in the inframammary fold incision group (44.8 ± 43.6 months), and 23.3% (50/216) in the single axillary or lateral chest incision (29.63 ± 20.6 months; nipple sensation: *P* = 0.04, time since surgery: *P* = 0.01, Table 2).

As for factors related to preserved nipple sensation, 133 (28.9%) grade III nipple sensation NSM procedures group were compared with 327 (71.1%) abnormal nipple sensation group (Table 3). The statistically significant factors for nipple-related sensation are age (50.4 ± 9 versus 47.5 ± 9, *P* < 0.01), tumor location (*P* = 0.03), different BMI categories (*P* = 0.04), different surgical methods (*P* = 0.01), axillary staging method (*P* = 0.04), surgical wound location (0.01), and follow-up duration (52.1 ± 32.4 versus 38.4 ± 31.8 months, *P* < 0.01). Statistically significant differences in skin sensation were found in different surgical methods (*P* < 0.01), surgical wound locations (*P* < 0.01), adjuvant radiotherapy (*P* = 0.01), and follow-up duration (48.5 ± 32.6 versus 39.2 ± 32.1 months, *P* < 0.01).

**Table 3:** Factors related to nipple and skin sensation post nipple-sparing mastectomy

	**Nipple sensation**				**Skin sensation (average value of 4 quadrants)**			
	Total *N* = 460	Abnormal *n* = 327	Normal (3) *n* = 133	*p*-value	Total *N* = 460	Abnormal *n* = 301	Normal (3) *n* = 159	*p*-value
**Age, y**								
**A**ll	48.4 ± 9.1	47.5 ± 9	50.4 ± 9	< 0.01	48.4 ± 9.1	47.9 ± 9	49.2 ± 9.1	0.17
< 40	75 (16.3)	58 (17.7)	17 (12.8)	0.33	75 (16.3)	49 (16.3)	26 (16.3)	0.53
≧ 40, < 60	329 (71.5)	232 (70.9)	97 (72.9)		329 (71.5)	219 (72.8)	110 (69.2)	
≧ 60	56 (12.2)	37 (11.3)	19 (14.3)		56 (12.2)	33 (10.9)	23 (14.5)	
**Location**				0.03				0.13
Right	239 (51.9)	180 (55.1)	59 (44.4)		239 (51.9)	164 (54.5)	75 (47.2)	
Left	221 (48.1)	147 (44.9)	74 (55.6)		221 (48.1)	137 (45.5)	84 (52.8)	
**BMI**								
**ALL**	23.3 ± 3.7	23.4 ± 3.8	22.9 ± 3.3	0.24	23.3 ± 3.7	23.3 ± 3.7	23.2 ± 3.7	0.73
< 18	19 (4.1)	18 (5.5)	1 (0.8)	0.04	19 (4.1)	17 (5.6)	2 (1.3)	0.07
18–24	271 (59)	186 (56.9)	85 (63.9)		271 (59)	172 (58)	99 (62.4)	
≧ 24	170 (36.9)	123 (37.6)	47 (35.3)		170 (36.9)	112 (36.4)	58 (36.3)	
**Surgical methods**				0.01				< 0.01
C-NSM	174 (37.8)	111 (33.9)	63 (47.4)		174 (37.8)	87 (28.9)	87 (54.7)	
E-NSM	195 (42.4)	143 (43.8)	52 (39.1)		195 (42.4)	137 (45.5)	58 (36.5)	
R-NSM	91 (19.8)	73 (22.3)	18 (13.5)		91 (19.8)	77 (25.6)	14(8.8)	
**Specimen size, g**								
**ALL**	327.1 ± 184.5	333.6 ± 185.8	311.3 ± 180.9	0.26	327.1 ± 184.5	327.4 ± 187.1	326.4 ± 179.9	0.95
< 180	66 (16)	42 (14.4)	24 (20)	0.47	66 (16.01)	41 (15.1)	25 (17.8)	0.76
180–320	173 (42)	122 (41.8)	51 (42.5)		173 (41.99)	119 (43.7)	54 (38.6)	
320–450	93 (22.6)	69 (23.6)	24 (20)		93 (22.57)	60 (22.1)	33 (23.6)	
> 450	80 (19.4)	59 (20.2)	21 (17.5)		80 (19.41)	52 (19.1)	28 (20.0)	
**Axillary staging method**				0.04				0.09
SLNB + ALND	85 (20.6)	71 (24.1)	14 (11.8)		85 (20.6)	59 (21.9)	26 (18.1)	
SLNB	298 (71.9)	204 (69.1)	94 (78.9)		298 (71.9)	197 (72.9)	101 (70.1)	
ALND	31 (7.5)	20 (6.8)	11 (9.2)		31 (7.5)	14 (5.2)	17 (11.8)	
**Tumor size, cm**	2.5 ± 2.2	2.6 ± 2.1	2.5 ± 2.5	0.78	2.5 ± 2.2	2.5 ± 2.1	2.7 ± 2.5	032
**Operation time**	224.2 ± 124.3	227.8 ± 125.9	215.4 ± 120.2	0.33	224.2 ± 124.3	223.5 ± 123.0	225.6 ± 127.1	0.86
**Reconstruction**				0.06				0.09
Yes	383 (83. 3)	279 (85.3)	104 (78.2)		383 (83.3)	257 (85.4)	126 (79. 3)	
No	77 (16.7)	48 (14.7)	29 (21.8)		77 (16.7)	44 (14.6)	33 (20.7)	
**Reconstruction method**				0.53				0.76
Implant	350 (91.9)	253 (91.3)	97 (93.3)		350 (91.9)	235 (92.2)	115 (91.3)	
Flap	31 (8.1)	24 (8.7)	7 (6.7)		31 (8.1)	20 (7.8)	11 (8.7)	
**Surgical wound location**				0.01				< 0.01
Inframammary fold	18 (3.9)	12 (3.7)	6 (4.5)		18 (3.9)	8 (2.7)	10 (6.3)	
Peri-areolar	156 (33.9)	96 (29.3)	60 (45.1)		156 (33.9)	78 (25.9)	78 (49.1)	
Single axillary or lateral chest	215 (47.8)	165 (50. 5)	50 (37.6)		215 (47.8)	172 (57.1)	43 (27.0)	
Upper outer (radial + other)	71 (15.4)	54 (16.5)	17 (12.8)		71 (15.4)	43 (14.3)	28 (17.6)	
**Blood loss**	65.6 ± 65.5	67.4 ± 69.7	61.2 ± 53.9	0.35	65.6 ± 65.5	64.1 ± 68.5	68.4 ± 59.6	0.51
**Complication**				0.26				0.59
Yes	102 (22.2)	77 (23.5)	25 (18.8)		102 (22.2)	69 (22.9)	33 (20.7)	
No	358 (77.8)	250 (76.5)	108 (81.2)		358 (77.8)	232 (77.1)	126 (79.3)	
**Nipple ischemia event**				0.27				
0	399 (86.7)	281 (86.2)	118 (88.0)					
1	29 (6.4)	19 (5.9)	10 (7.5)					
2	32 (6.9)	26 (7.9)	6 (4.5)					
**Chemotherapy**				0.11				0.6
Yes	174 (40.6)	130 (42.4)	44 (36.1)		174 (40.6)	110 (39.1)	64 (43.2)	
No	255 (59.4)	177 (57.6)	78 (63.9)		255 (59.4)	171 (60.9)	84 (56.8)	
**Radiotherapy**				0.16				0.01
Yes	116 (27.1)	77 (25.1)	39 (31.9)		116 (27.1)	68 (24.2)	48 (32.4)	
No	313 (72.9)	230 (74.9)	83 (68.1)		313 (72.9)	213 (75.8)	100 (67.6)	
**Follow-up time (time since surgery) months**	42.3 ± 32.5	38.4 ± 31.8	52.1 ± 32.4	< 0.01	42.3 ± 32.5	39.2 ± 32.1	48.5 ± 32.6	< 0.01

*NSM* nipple-sparing mastectomy, *C-NSM* conventional NSM, *E-NSM* endoscopic-assisted NSM, *R-NSM* robotic-assisted NSM, *SD* standard deviation, *NAC* nipple areolar complex, *BMI* body mass index, *ALN* axillary lymph node, *SLNB* sentinel lymph node biopsy, *ALND* axillary lymph node dissection

Of these 460 NSM procedures, 29 (6.4%) had transient nipple ischemia and 32(6.9%) with partial nipple necrosis. There was no direct correlation between nipple ischemia/necrosis and subsequent nipple sensation. Possible factors of nipple or skin sensation were further validated in univariate and multivariate analysis (Table 4). In univariate analysis, surgical methods, wound location, and follow-up time were noted as significant risk factors. In multivariate analysis, longer follow-up time showed significantly improved nipple or skin sensation (> 60 versus ≦ 12 months: nipple odds ratio (OR) = 5.75, *P* < 0.01; skin: *OR* = 1.97, *p* < 0.05). Peri-areolar wound location showed higher nipple sensation (*OR*: 2.1, *P* = 0.02), while single axillary or lateral chest incision showed decreased skin sensation (*OR*: 0.4, *P* < 0.01) when compared with the upper outer quadrant incision (Table 4). The R-NSM group showed decreased nipple (*OR* = 0.4, *P* < 0.01) or skin (*OR* = 0.16, *P* < 0.01) sensation, and E-NSM group also had lower skin sensation (*OR* = 0.42, *P* < 0.01) compared with C-NSM group.

**Table 4:** Univariate and multivariate analysis of factors related to nipple or skin sensation post nipple-sparing mastectomy

**Univariate analysis**									
				**Nipple sensation**			**Skin sensation**		
	Total	Satisfy^a^ (*N*)	%	*OR*	*CI*	*p*-value	*OR*	*CI*	*p*-value
**Age**									
< 40	**75**	**17**	**22.7**	1			1		
≧ 40 and < 60	329	97	**29.5**	1.42	0.79–2.57	0.23	0.94	0.55–1.60	0.83
≧ 60	56	19	**33.9**	1.75	0.80–3.79	0.15	1.31	0.64–2.68	0.45
**Surgical methods**									
C-NSM	174	63	**36.2**	1			1		
E-NSM	195	52	**26.7**	0.64	0.41–0.99	0.04	0.42	0.27–0.61	< 0.01
R-NSM	91	18	**19.8**	0.43	0.23–0.79	< 0.01	0.18	0.09–0.34	< 0.01
**Surgical ALN staging method**									
SLNB	**298**	**94**	**31.5**	1			1		
SLNB + ALND	85	14	**16.5**	0.42	0.22–0.79	< 0.01	0.85	0.51–1.44	0.56
ALND	31	11	**35.5**	1.19	0.54–2.59	0.65	2.36	1.12–4.99	0.02
**Surgical wound location**									
Upper outer (radial + other)	**71**	**17**	**23.9**	1			1		
Infra-mammary Fold	18	6	**33.3**	1.58	0.51–4.87	0.41	1.91	0.67–5.45	0.22
Peri-areolar	156	60	**38.5**	1.98	1.05–3.74	0.03	1.53	0.86–2.71	0.14
Single axillary or lateral chest	215	50	**23.3**	0.96	0.51–1.8	0.90	0.38	0.40–0.68	< 0.01
**Specimen size, g**									
< 180	**66**	**24**	**36.4**	1			1		
180–320	173	51	**29.5**	0.73	0.40–1.33	0.30	0.74	0.41–1.34	0.32
320–450	93	24	**25.8**	0.60	0.30–1.20	0.15	0.90	0.46–1.73	0.75
> 450	80	21	**26.3**	0.62	0.30–1.26	0.18	0.88	0.44–1.73	0.71
**Operation time**				0.99	0.99–1.00	0.33	1.00	0.99–1.00	0.86
**Follow-up time (time since surgery), month**									
≦ 12 m	86	10	**11.6**	1			1		
> 12 m and ≦ 36 m	155	39	**25.2**	2.55	1.2–5.42	0.01	1.52	0.83–2.79	0.17
> 36 m and ≦ 60 m	97	37	**38.1**	4.68	2.15–10.1	< 0.01	2.12	1.11–4.05	0.02
> 60 m	122	47	**38.5**	4.76	2.24–10.1	< 0.01	2.45	1.32–4.53	< 0.01
**Multivariate analysis**									
				**Nipple sensation (stepwise)**			**Skin sensation (stepwise)**		
	Total	Satisfy (*N*)	%	*OR*	*CI*	*p*-value	*OR*	*CI*	*p*-value
**Surgical methods**									
C-NSM	174	63	**36.2**	1			1		
E-NSM	195	52	**26.7**	0.39	0.19–0.79	0.01	0.58	0.31–1.08	0.08
R-NSM	91	18	**19.8**	0.23	0.09–0.6	< 0.01	0.29	.011–0.74	0.01
**Surgical wound location**									
Upper outer (radial + other)	**71**	**17**	**23.9**	1			1		
Inframammary fold	18	6	**33.3**	2.08	0.62–6.88	0.23	2.02	0.69–5.95	0.19
Peri-areolar	156	60	**38.5**	2.84	1.4–5.73	< 0.01	1.74	0.95–3.22	0.07
Single axillary or lateral chest	215	50	**23.3**	3.76	1.32–10.7	0.01	0.79	0.32–1.97	0.61
**Follow-up time (time since surgery), month**									
≦ 12 m	86	10	**11.6**	1			1		
> 12 m and ≦ 36 m	155	39	**25.2**	2.77	1.28–5.98	< 0.01	1.96	1.03–3.75	0.04
> 36 m and ≦ 60 m	97	37	**38.1**	5.49	2.46–12.3	< 0.01	2.72	1.35–5.48	< 0.01
> 60 m	122	47	**38.5**	5.75	2.55–12.9	< 0.01	1.97	1.0–3.89	< 0.05
**Multivariate Analysis**									
				**Nipple sensation (stepwise)**			**Skin sensation (stepwise)**		
	Total	Satisfy (N)	%	*OR*	*CI*	*p*-value	*OR*	*CI*	*p*-value
**Surgical wound location**									
Upper outer (radial + other)	**71**	**17**	**23.9**	1			1		
Inframammary fold	18	6	**33.3**	1.8	0.56–5.86	0.31	1.9	0.65–5.5	0.23
Peri-areolar	156	60	**38.5**	2.1	1.09–4.04	0.02	1.5	0.84–2.67	0.16
Single axillary or lateral chest	215	50	**23.3**	1.2	0.58–2.32	0.65	0.4	0.19–0.67	< 0.01
**Follow-up time (time since surgery), month**									
≦ 12 m	86	10	**11.6**	1			1		
> 12 m and ≦ 36 m	155	39	**25.2**	2.5	1.2–5.53	0.01	1.8	0.97–3.5	0.06
> 36 m and ≦ 60 m	97	37	**38.1**	4.8	2.19–10.5	< 0.01	2.4	1.2–4.71	0.01
> 60 m	122	47	**38.5**	4.2	1.97–9.32	< 0.01	1.7	0.86–3.18	0.12
**Multivariate analysis**									
				**Nipple sensation (stepwise)**			**Skin sensation (stepwise)**		
	Total	Satisfy (*N*)	%	*OR*	*CI*	*p*-value	*OR*	*CI*	*p*-value
**Surgical methods**									
C-NSM	174	63	**36.2**	1			1		
E-NSM	195	52	**26.7**	0.7	0.41–1.04	0.07	0.42	0.27–0.65	< 0.01
R-NSM	91	18	**19.8**	0.4	0.21–0.77	< 0.01	0.16	0.08–0.32	< 0.01
**Follow-up time after op, m**									
≦ 12 m	86	10	**11.6**	1			1		
> 12 m and ≦ 36 m	155	39	**25.2**	2.9	1.35–6.2	< 0.01	1.9	1.05–3.72	0.03
> 36 m and ≦ 60 m	97	37	**38.1**	5.4	2.4–11.9	< 0.01	2.7	1.4–5.55	< 0.01
> 60 m	122	47	**38.5**	4.5	2.1–9.64	< 0.01	2.2	1.2–4.26	0.01

^a^Satisfy: preserved nipple sensation

The results of the current study were summarized and compared with other reported series in the literature as shown in Table 5.

**Table 5:** Literature review of nipple areolar complex sensation after nipple-sparing mastectomy

Reference					Sensation at skin, *N* (%)/score				
Authors	Journal	Publish year	Number	Sensation at nipple, *N* (%)/score	Upper lateral quadrant	Upper medial quadrant	Lower lateral quadrant	Lower medial quadrant	Follow-up times
Djohan et al. [1]	*Plastic and Reconstructive Surgery*	2010	77	19 (15.3%)	N/A				23 months
Kenji Yano et al. [20]	*Annals of Plastic Surgery*	2011	43	4.17 (g)	3.69 (g)				12–61 months, mean: 31 months
Rodriguez-Unda et al. [23]	Annals of Plastic Surgery	2014	12	44.5 (g/mm^2^)	49.2 (g/mm^2^)	36.7 (g/mm^2^)	73.6 (g/mm^2^)	47.8 (g/mm^2^)	31.6 months, mean
Lesly A. et al. [13]	*Journal of Surgical Oncology*	2016	33	0.125	0.75 (filament diameters: 0 = no sensation, 1 = 6.65)				Minimum of 12 months
Norbert et al. [21]	*Clin Hemorheol Microcirc*	2017	10	8.8 (g)	2.6 (g)				684 days in average
Prakasit et al. [18]	Plastic and Reconstructive Surgery	2018	35	13 (37%)	15 (43%)	16 (46%)	16 (46%)	17 (49%)	Mean 24 months (range 2–104 months)
Kim et al. [22]	*Aesthetic Plastic Surgery*	2018	55	2.12 ± 0.58 (NAC sensitivity score)	N/A				At least 3 months
Kristina et al. [19]	*The Breast Journal*	2019	40	14 (35%)	N/A				1–3 years
Jian Farhadi et al. [9]	*Journal of Reconstructive Microsurgery*	2020	59	N/A	49 (83%)	51 (86%)	46 (77%)	48 (81%)	23.4 ± 11.1 months
Lai et al	Current study	2023	460	2.1 ± 0.7 (nipple sensation average value: mean, SD)	Lateral 2 ± 0.8	Upper 2.1 ± 0.8	Inferior 2.1 ± 0.8	Medial 2.1 ± 0.8	42.4 ± 32.5 months

## Discussion

In this current study, 460 cases NSM procedures from Aug 2011 to April 2022 were enrolled and with clear evaluations of the impact of time lapses on the recovery of nipple or skin sensation. The mean follow-up duration of 42.4 ± 32.5 months (ranged: 1–143) was adequate to evaluate the recovery of skin or nipple sensation. In all cases, the sensation assessment evaluation uses “five areas measurement points” (Fig. 2). With 230 cases having more than one measurement time (some patients even had more than five times evaluated throughout the follow-up), 28.9% (133/460) and 34.5% (159/460) of the NSM procedures retained nipple sensations and skin sensations (grade III), respectively.

Recent studies on the incidence of nipple and skin sensation loss or recovery have varied and inconclusive results due to different maneuvers used and timing of sensation assessment among different reported studies (Table 5). The reported nipple sensation ranged from 10 to 75% (Table 5) [7–9, 19, 23–25]. In general, the skin had superior filament discrimination compared to the NAC. A study evaluating 150 healthy women regarding normal breast sensitivity found that the skin of the superior quadrant was the most sensitive part of the breast, followed by the areola, and the least sensitive part was the nipple [26]. Nonetheless, we found that the mean average grade score of breast skin sensation post-NSM over four quadrants was 2.1 ± 0.7, which was similar to the nipple sensation (2.1 ± 0.7, Table 2) in our study.

From univariate and multivariate analysis in our study, factors related to nipple or skin sensation were surgical approaches, wound incision types, and long follow-up duration (Tables 3 and 4). In a recently published RCT [14], R-NSM was associated with better preserved nipple sensation than C-NSM (31.6% versus 0%, *P* = 0.0002). In the current study, the preserved (grade III) nipple sensation rate was 36.2% (63/174) in the C-NSM group (mean follow up time 45.6 ± 34 months), 26.7% (52/195) in the E-NSM group (44.7 ± 35.8 months), and 19.7% (18/91) in the R-NSM (31.8 ± 16 months) group (nipple sensation: *P* = 0.06, follow up duration: *P* < 0.01 (C-NSM or E-NSM versus R-NSM), Table 2). In multivariate analysis, E-NSM or R-NSM showed decreased nipple or skin sensation if compared with C-NSM (Table 4). For patients who received R-NSM or E-NSM (since 2014) procedures, single axillary (or lateral chest incision) was the most frequently adopted incision type, however, which was associated with significantly decreased skin sensation. Peri-areolar incision showed significantly better nipple sensation than upper outer incision (Tables 3 and 4), which was rarely reported before, if any.

“Time from surgery to last evaluation” was a significant factor associated with sensation recovery or preservation in nipple (52.1 ± 32.4 versus 38.4 ± 31.8, *P* < 0.01) or skin sensation (48.5 ± 32.6 versus 39.2 ± 32.1, *P* < 0.01) post NSM. In multivariate analysis, follow-up duration is a significant factor in preserving nipple (*OR* = 5.75, *P* < 0.01) or skin (*OR* = 1.97, *P* < 0.05) sensation. In subgroup analysis of different surgical methods on the impact of nipple or skin sensation, there was significantly longer follow-up duration in C-NSM group (45.6 ± 34 months) or E-NSM (44.7 ± 35.8 months) than R-NSM (31.8 ± 16) group (*P* < 0.01, Table 2). The concept of “time since surgery” is associated with improved nipple sensation. Rodriguez-Unda et al. [27] suggested that factors such as age, time since surgery, and surgical approach have been linked to nerve regeneration. Shridharani et al. [28] showed that the sensory nerve of the nipple and skin flap “need time” to reinnervation. The actual time needed was unclear; however, it showed progressive improvement of constant touch over time with a lag period of the first 12 months. It seems that it takes 18 to 24 months of sensory recovery after breast reconstruction. These assumptions were consistent with our findings of follow-up time and nipple sensation in Table 4.

The sensory nerves of the NAC and breast skin come from lateral and cutaneous branches of the 3rd–5th intercostal nerves (mainly the 4th lateral cutaneous branch). The lateral cutaneous branches were a greater branch that took a deep course from muscle penetrating breast parenchyma and pierced to the posterior surface of nipple [29–33]. Montagne and Macpherson et al. [34] demonstrated that the neural elements concentrate at the base of the nipple, with few at the side of the nipple and even fewer in the areolar. Therefore, it is unsurprising how nipple and skin sensations were lost or diminished after NSM [35, 36], especially when retro-areolar tissue sampling was performed for intraoperative frozen section.

In the current study, nipple and skin flap sensation differed according to different surgical incisions and operation methods, which was rarely discussed before. E-NSM or R-NSM did not showed improved nipple or skin sensation than C-NSM even in multivariate analysis (Tables 2, 3 and 4). Skin incision and types of operations were highly correlated. A single axillary or lateral chest incision, which was frequently the preferred incision in E-NSM or R-NSM procedures, and upper-outer, inframammary, or peri-areolar incisions were frequently used by C-NSM procedures (Fig. 3). In multivariate analysis, peri-areolar incision showed significantly higher nipple sensation preservation, while singe axillary or lateral chest incision showed significantly decrease skin sensation (Table 4). Based on the anatomic basis of nerve supply, the single axilla or lateral chest incision, which started dissection from a “lateral to medial” fashion with incision lateral to the lateral border of the breast, might increase the risk of injury of the lateral cutaneous branches of 4th intercostal nerve when coursing into the breast [19]. The dissection plane of peri-areolar, inframammary, or upper outer incision started “medial to lateral” and usually stopped at the lateral border of the breast, which might help in preserving the entry of the lateral cutaneous branch of 4th intercostal nerve and therefore led to higher preservation of skin sensation.

The peri-areolar incision was associated with higher nipple sensation in multivariate analysis (Table 4). In our current study, sub-nipple samplings were routinely performed to prevent occult cancer left behind NAC. Peri-areolar incision was associated with higher risk of NAC ischemia necrosis, and surgeons tend to leave thicker skin flap to avoid over-thinning of retro-areolar tissue and subsequent NAC total necrosis. However, in E-NSM or R-NSM, the risk of total NAC necrosis was decreased due to the placement of skin incision far away from NAC, and usually, the skin flap beneath NAC was thinner due to oncologic safety consideration. These unique technical differences might have contributed to the observation of higher nipple sensation preservation in peri-areolar incision (frequent used in C-NSM) than single axilla or lateral chest incision (usually used in E-NSM or R-NSM, Fig. 3, Tables 3 and 4).

In some studies, there was hypothesis that the factors contributing to nipple necrosis might be correlated with loss of nipple sensation as well owing to the virtue of related neurovascular bundle anatomy when dissecting the superficial breast and skin fascia [20, 21, 37]. However, we found no correlations between NAC ischemia necrosis grading and nipple or skin sensation post-NSM (Table 3). Post-mastectomy radiotherapy (PMRT) was indicated in patients with poorer prognostic factors, like tumor size > 5 cm or lymph nodes metastasis, to decrease disease recurrence and prolonged survival [22]. There was 27% of NSM procedures in the current cohort who received PMRT, but these were not associated with lower preservation of nipple sensation. In contrast, we observed a higher preserved skin sensation in patients who received PMRT (41.3% versus 31.9%, *P* = 0.01). These findings were consistent with Khan et al. [7], which showed that PMRT did not deteriorate nipple or skin sensation.

In our current study, the focus is on nipple and skin sensation post NSM, and the authors successfully identified factors associated with preserved nipple or skin sensation with further validation of the relationship between nipple or skin sensation recovery to the duration of “time since surgery.” Our study is limited due to its retrospective nature, small sample size, and possible selection bias. Furthermore, the results of nipple or skin sensation test were subjective and not equally measured within a specific time period. However, our study enrolled a large number of NSM cases with detailed clinicopathologic, perioperative parameters, and different surgical approaches or incisions to enable us to have a comprehensive analysis of possible factors related to preserved nipple or skin sensation post NSM. A long-term follow-up of mean 42.4 ± 32.5 months also enabled us to show gradual recovery of nipple or skin sensation with different “time after surgery” period. Therefore, the results and information derived from the current study are valuable.

## Conclusion

In our current study, the authors successfully demonstrated strong evidence that a significant improvement of nipple and skin sensation in patients who received NSM had to do with a longer “time after surgery” period. The results derived from this study will thus enable better discussion with patient regarding the impact of different operations methods and skin incision on skin and nipple sensation. This is definitely valuable in improving patients’ outcomes and QoL following NSM, especially in an era of shared decision-making prior to surgery.

## Data Availability

The data used in the current study could be provided by request to the principal investigator HWL after acceptance of the manuscript.

## Abbreviations

NSM: Nipple-sparing mastectomy
C-NSM: Conventional NSM
E-NSM: Endoscopic-assisted NSM
R-NSM: Robotic-assisted NSM
NAC: Nipple areolar complex
OR: Odds ratio
SSM: Skin-sparing mastectomy
CCH: Changhua Christian Hospital
IRB: Institutional review board
QoL: Quality of life
